# Chimeric Antigen Receptors Expand the Repertoire of Antigenic Macromolecules for Cellular Immunity

**DOI:** 10.3390/cells10123356

**Published:** 2021-11-30

**Authors:** John T. Keane, Avery D. Posey

**Affiliations:** 1 Department of Systems Pharmacology and Translational Therapeutics, Perelman School of Medicine, University of Pennsylvania, Philadelphia, PA 19104, USA; john.keane@pennmedicine.upenn.edu; 2 Corporal Michael J. Crescenz VA Medical Center, Philadelphia, PA 19104, USA

**Keywords:** CAR T-cells, immunity, cancer

## Abstract

T-cell therapies have made significant improvements in cancer treatment over the last decade. One cellular therapy utilizing T-cells involves the use of a chimeric MHC-independent antigen-recognition receptor, typically referred to as a chimeric antigen receptor (CAR). CAR molecules, while mostly limited to the recognition of antigens on the surface of tumor cells, can also be utilized to exploit the diverse repertoire of macromolecules targetable by antibodies, which are incorporated into the CAR design. Leaning into this expansion of target macromolecules will enhance the diversity of antigens T-cells can target and may improve the tumor-specificity of CAR T-cell therapy. This review explores the types of macromolecules targetable by T-cells through endogenous and synthetic antigen-specific receptors.

## 1. Introduction

T-lymphocytes (or T-cells) are the principal effectors of the human immune system, in charge of effects ranging from B-cell licensing for antibody production to direct cytolytic activity. The major function of T-cells is based on interactions between the T-cell receptor (TCR) and a cognate peptide within a major histocompatibility complex (MHC) molecule on either infected/malignant cells or professional antigen presenting cells [1].

Recently, T-cells have been at the forefront of new cancer treatments, especially the advent of engineered cellular immunotherapies including chimeric antigen receptor (CAR) T-cell therapy [2]. One advantage of CAR T-cells is the ability to recognize antigens expressed on the cell surface without the need for antigen presentation by MHC molecules, reducing the need to consider limitations of histocompatibility and variability of peptide-MHC presentation. CAR-based targeting is generally directed through the antigen-recognizing features of a single chain variable fragment (scFv) domain presented on the surface of CAR T-cells. This scFv allows CAR T-cells to bind to and target any cell surface macromolecule defined by antibody binding. To complete this receptor, the scFv is molecularly fused to a transmembrane domain that connects the antigen-recognizing extracellular domain to intracellular signaling and activation by the CD3ζ activation domain derived from the TCR complex. The first generation of CAR T-cells utilized only the CD3ζ domain. However, future generations of CAR T-cells built on the intercellular signaling by using costimulatory domains, such as CD28 or 4-1BB, to enhance the stimulation of the CAR T-cells. Second generation CAR T-cells incorporate a single costimulatory domain into the CAR molecule, while third generation CAR T-cells utilize use multiple costimulatory domains in tandem [3]. Recent advances in technology have led to the generation of fourth generation CAR T-cells, nicknamed TRUCKs (T cells redirected for antigen-unrestricted cytokine-initiated killing) [4]. Fourth generation CAR T-cells are capable of constitutively or inducibly secreting pro-inflammation factors, such as cytokines, that may promote persistence or function. CAR molecules and additional effector molecules have conventionally been introduced into T-cells through viral transduction utilizing lentivirus or retrovirus, transposition using Sleeping Beauty or PiggyBac transposases, or transient transfection through mRNA delivery. However, recent advances employing homology-directed recombination with CRISPR [5,6,7] and other gene-editing tools allows site-specific integration of new genetic material [8]. This advance will decrease the batch-to-batch variability of manufactured CAR T-cell products and may elucidate specific loci or safe harbors for integration that improve clinical efficacy.

**Figure 1:**
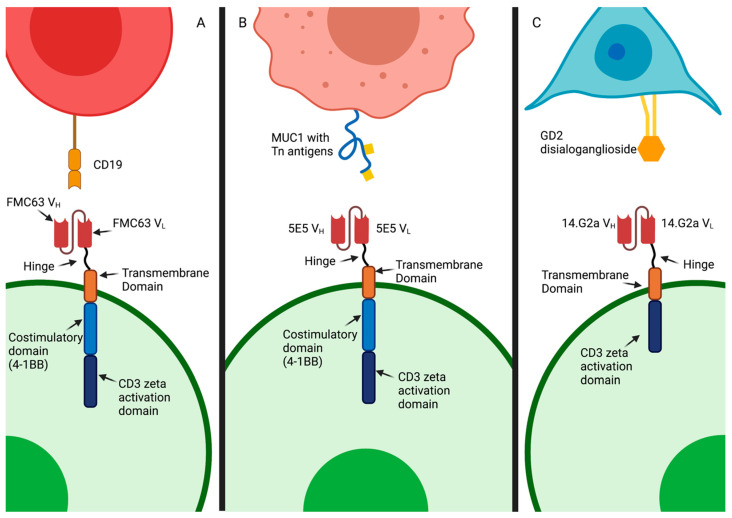
Schematic representation of exemplary CAR T-cell interactions with antigenic targets made of amino acids, such as FMC63-based anti-CD19 CAR T-cells targeting CD19+ B-cells (**A**), 5E5-based anti-Tn-MUC1 CAR T-cells targeting the glycoepitope Tn-MUC1 on a solid tumor cell (**B**), and 14G2a-based anti-GD2 CAR T-cells targeting the ganglioside GD2 on a neuroblastoma cell (**C**).

CAR T-cells are under development to target many forms of cancer, with the best clinical results demonstrated thus far against hematological disorders, such as B-cell leukemia and lymphoma through the targeting of the lineage-restricted B-cell molecule CD19 and multiple myeloma by targeting B-cell maturation antigen (BCMA). Figure 1 shows three clinical-stage CAR T-cell therapies and their target antigens.

This review focuses on the types of macromolecules targetable by endogenous T-cells and how CAR T-cells expand this repertoire of cellular immunity antigens.

## 2. Peptides

T-cells recognize short chains of amino acids, called peptides, through engagement of the TCR with cognate peptide-MHC complexes. The TCR is composed, in part, of a heterodimer of variable α- and β-chains and can bind residues found within the MHC molecules as well as the peptide. This interaction restricts antigenic recognition by T-cells almost solely to the amino acids found within the MHC complex [9]. There are two types of MHCs: class I and class II. TCR recognition of peptides found within MHC class I molecules require the CD8 co-receptor and induces activation of CD8+ T-cells, influencing cytotoxic T cell responses. MHC class II molecules are expressed by antigen presenting cells (APCs), require the CD4 co-receptor for TCR engagement, and these interactions typically lead to the activation of helper T-cells responses.

In addition to the αβ TCR heterodimers, the TCR complex also includes the γ, δ, ε, and ζ subunits of CD3, which induce signal transduction upon engagement of TCR and cognate peptide-MHC [10]. A TCR is assembled through a large set of gene segments in a process known as V (variable) D (diversity) J (joining) recombination [11]. The recombination process involves double-stranded DNA nicking, gene segment excision, and ligation of remaining gene segments into the coding sequences of functioning TCRs. This process has the ability to produce more than 10^15^ possible TCRs with highly variable antigen recognition [12], which allows T-cells to recognize an immensely broad and diverse repertoire of peptide-MHC.

MHC class I molecules are composed from a heavy chain as well as a β_2-_microgobulin. There are three polymorphic genes in humans that encode the MHC class I heavy chains of HLA-A, HLA-B, and HLA-C, which leads to over 200+ variants of HLA-A, 500+ variants of HLA-B and 100+ variants of HLA-C genes [13]. These MHC heavy chain polymorphisms are responsible for the generation of divergent peptide binding grooves and unique collections of MHC-presented peptides, which complicates the adoptive transfer of T-cells or specific TCR genes from individual-to-individual (as a universal cancer treatment, for instance) due to incomplete histocompatibility [14]. Other MHC-related factors that can limit T-cell recognition of antigenic peptides is MHC class I downregulation, impairment of antigen presentation machinery, and rare or absent MHC class I presentation of mutated peptides. In papillary thyroid cancer, for example, downregulation of MHC class I has been shown to influence a decrease in the number of tumor-infiltrating lymphocytes (TILs) found in the tumor and is associated with worse clinical outcome [15].

A major limitation for antigenic recognition by CAR molecules is the requirement for cell surface or extracellular presentation. As such, the number of antigens that can be targeted by CARs is reduced by many orders of magnitudes when compared to the quantity of antigens T-cells can recognize through TCR engagement with peptide-MHC molecules. However, the generation of “TCR-like antibodies”, a class of antibodies able to recognize minor histocompatibility antigens (mHAgs) with an affinity 10^3^–10^5^ times higher than natural TCR binding [16], may endow CAR T-cells with the ability to recognize specific MHC-bound antigens, including intracellular targets. A study from Walseng et al. developed a “TCR-CAR” against peptide fragments of MART-1 (DMF5 scFv) and TGFβR2 (Radium-1) [17]. These TCR-CAR molecules redirected T-cells and natural killer cells, represented by the NK-92 cell line, toward the target epitope of the two genes. The CAR T-cells and NK cells were able to clear cells presenting either MART-1 or TGFbR2 peptides within their MHC complexes. TCR-CARs have also been generated to selectively target a peptide comprised of amino acids 235–243 of Wilm’s tumor-1 (WT1), an antigen found overexpressed in leukemia, lymphoma, as well as solid tumors, when presented by the MHC cleft of HLA-A*2402 [18].

Perhaps the most successful CAR molecule is the anti-CD19 CAR, especially in the context of CAR engineered T-cells (CART19). CD19 is a lineage-restricted B cell molecule that is expressed on both healthy and malignant B cells. As early as 2011, research has shown the success of CART19 in patients with B-cell cancers. In a pilot clinical trial, three patients with chemotherapy-resistant chronic lymphocytic leukemia were treated with CART19 cells; two patients attained a complete remission and a third patient achieved a partial remission [19]. In contrast to peptide-MHC recognition, CART19 cells bind to and are activated by CD19 expressed on the surface of B-cells, independent of MHC recognition, coreceptor engagement, and antigen presentation machinery. CART19 has shown remarkable and reproducible success in clinical trials for patients with B-cell leukemia and lymphoma and four FDA approved CD19-targeted CAR T-cell therapies are approved to date: (in order of first approval) tisagenlecleucel, axicabtagene ciloleucel, brexucabtagene autoleucel, and lisocabtagene maraleucel. All four approved CD19-targeted CAR therapies utilize an scFv from the anti-CD19 antibody FMC63. Idecabtagene vicleucel is another CAR T-cell therapy targeting BCMA for relapsed/refractory multiple myeloma that was also FDA approved in 2021.

A budding area of CAR T-cell development is redirecting T-cell specificity towards antigens in the extracellular environment, in contrast to targeting cell surface antigens. CAR T-cells targeting soluble TGF-β, an immunosuppressive cytokine expressed by many solid tumors, expanded many fold in response to TGF-β stimulation, while non-specific CAR T-cells exhibited very low persistence due to the inhibitory effects of the suppressive cytokine [20]. Although anti-TGF-β CAR T-cells were not cytolytic, this approach demonstrated that CARs can switch immunosuppressive factors produced by tumors into immunostimulatory signals. While this therapy converts an immunosuppressive factor into an immune stimulator, there may be some concerns about the developmental changes in T-cells, as TGF-β promotes developmental changes in CD8+ T-cell differentiation [21]. More studies and trials are needed to determine if this will have any negative effects on the immune system.

Another unique class of targets for CAR T-cells may exist within the structural components of the extracellular matrix (ECM). Wagner et al. generated CAR T-cell that can target extra domain B (EDB) of fibronectin, a splice variant of fibronectin produced by many types of solid tumors [22]. Targeting the ECM of multiple human solid tumors with anti-fibronectin CAR T-cells led to control of tumor growth and increased survival in several cell-line derived xenograft models. Additionally, the EDB domain of murine fibronectin was targeted by CAR T-cells constructed with a single-domain antibody (VHH) specific for EIIB [23]. In this model, B16 melanoma growth was slowed compared to control CAR T-cells, which improved T-cell infiltration and likely skewed an immunosuppressive TME towards an inflammatory TME.

## 3. Lipids

As described above, MHC class I and class II molecules present peptides on the surface of cells for recognition by the TCRs of T-cells. A third molecule, CD1, is used similarly by cells. However, while MHC complexes present peptides, CD1 presents lipids, including, but not limited to, glycolipids [24,25,26]. This difference is due to the hydrophobicity of the CD1 binding groove, which allows the presentation of hydrophilic elements of antigens to the CD1 protein [27]. Humans have five different CD1 isoforms that present lipids in different manners. These isoforms are CD1a, CD1b, CD1c, CD1d, and CD1e. The CD1 complexes present antigens to NKT-cells, which are restricted to the CD1 domain and are unable to recognize peptide-MHC [28].

CD1 complexes are similar in structure to MHC complexes with a heavy chain extracellular domain that binds to a β2microgobulin. However, when mouse CD1d proteins were crystallized, they showed a larger binding groove created with non-polar residues in which lipids could bind [27]. Another major difference between MHC complexes and CD1 complexes is the diverse array of molecules presented. MHC complexes, as stated above, are highly polymorphic, which allows for varying structures of restricted presentation. CD1 complexes, however, are able to bind wide arrays of different lipid molecules because this process does not require perfect positioning of the lipid molecules, thereby allowing CD1 complexes to bind multiple molecules with less restriction [29].

CD1-restricted T-cells contain a combination of αβ and γδ TCRs. They vary from MHC restricted T-cells by using less V_β_ genes that give rise to rearranged TCRβ chains alongside invariant TCRα arrangements [30]. This subset of cells is commonly referred to as natural killer T-cells (NKT-cells) due to the unique expression of CD161, a marker typically only found on NK cells [31]. NKT-cells are functionally very different depending on their life stage and is typically defined by whether or not the cell expresses CD4, with CD4+ NKT-cells exhibiting less differentiation than CD4− NKT-cells [32]. The maturation of these cells from CD4+ to CD4− is marked by an increase in the secretion of T_H_1 cytokines over T_H_2 cytokines. Due to this change, CD4− cells are more cytolytic than the CD4+ counterparts.

In cancer, CD1-restricted NKT-cells do not always have a positive effect. While exogenous treatment and activation of CD1-restricted NKT-cells by α-galactosylceramide (α-GalCer) has shown that CD1 restricted T-cells can have antitumor effect [33], these cells often do not naturally show cytotoxic activity against solid tumors without exogenous activation. In fact, NKT production of IL-13 has been known to act as an immune suppressant to CD8+ T-cells, which can impair immune anti-tumor activity [34]. However, other studies have shown that IFNγ production by circulating NKT-cells is important in the innate antitumor response [35] and an increased frequency of NKT-cells in blood or tumors can lead to favorable clinical outcomes in cancer patients [36]. The seemingly contradictive nature of NKT-cell activity could be due to the aforementioned differences in NKT-cell phenotypes.

The field of CAR T-cells targeting lipids has mostly focused on the ganglioside GD2, which is highly overexpressed in neuroblastoma and other solid tumors [37]. The advantage of GD2-targeting CAR T-cells is their ability to cross the blood brain barrier, which is an improvement over other forms of treatment, such as monoclonal antibodies for GD2. The first GD2-specific CAR T-cells were produced in 2009 to target cutaneous melanoma [38]. An scFv derived from the GD2-targeting antibody 14g2a was included in a CAR molecule costimulated by the intracellular signaling domains CD28 and OX40. GD2-specific CAR T-cells were able to kill a GD2+ mesenchymal stem cell (MSC) line without clearance of an isogenic GD2- MSC line, demonstrating antigenic specificity. In neuroblastoma, anti-GD2 CAR T-cells have been shown to control tumor growth effectively in mouse studies [39,40], and more recently in clinical studies. The first clinical study of GD2-specific CAR T-cells evaluated the safety of a first-generation CAR, which contains the intracellular domain of CD3ζ without a costimulatory domain, in contrast to the CAR designs discussed above, in Epstein-Barr virus-specific T-cells [41]. The persistence of GD2-specific CAR T-cells was observed for longer than six weeks and there was a correlation of CAR T-cell persistence and clinical response, including two complete remissions of neuroblastoma. In addition to GD2-specific CAR T-cells, CARs have also been developed to target the gangliosides O-acetyl-GD2, Neu5Gc-GM3, and GD3 as well as the globosides GloboH and SSEA4 [42].

Another approach to targeting the lipid framework is the targeting of the CD1 complex itself as opposed to a specific lipid within the molecule [43]. This work is being done in T-cell acute lymphoblastic leukemia, a disorder difficult to target with CAR T-cells due to shared markers on both effector CAR cells as well as malignant T cells. Cortical T-ALL (coT-ALL) is characterized by the surface expression of CD1a, a CD1 isoform only present on normal tissues during development of cortical thymocytes and in Langerhans cells. These CAR T-cells were able to specifically bind coT-ALL without any binding of non-malignant T cells. The CD1a CAR T-cells were able to eliminate T-ALL cell lines both in vitro and in vivo in preclinical studies. Fetal thymocytes were preserved throughout a coculture with the CD1a CAR T-cells, suggesting that this CAR T therapy may not pose a risk of thymic ablation.

## 4. Glycans

Glycans are mono- and polysaccharides produced by complex biosynthetic pathways that post-translationally modify proteins, lipids and nucleic acids with the involvement of nucleotide sugars as donors, while also mediating biological functions, such as protein folding, energy storage and metabolism, among other functions. One class of glycans, known as zwitterionic polysaccharides (ZPS), can activate the immune system through presentation on peptides bound by MHC class II molecules [44]. These molecules can be recognized by CD4+ T-cells, leading the formation of a memory immune response. ZPS have alternating positive and negative charge centers within the repeating units [45]. These structures often form during bacterial infections, such as the capsule of *B. fragilis* as well as the type 1 *S. pneumoniae* polysaccharide capsule. In addition to these, researchers have studied a ZPS known as polysaccharide A (PSA), expressed by a gram-negative bacterium *Bacteroides fragilis* [46]. This bacterium is symbiotic with the immune system, and studies have shown that germ-free mice expressing PSA on *B. fragilis* were able to maintain a healthy amount of CD4+ T-cells in the spleen as compared to WT mice [47]. A study by Cobb et al. [19] has shown that while all types of polysaccharides are able to be trafficked into APCs, such as dendritic cells, only those that are zwitterionic have the ability to colocalize with MHC II on the surface of the APCs.

Despite a consensus that most TCR and peptide-MHC interactions are glycan-independent, specific glycopeptides have been included as targets of vaccines. MUC1 is a membrane bound mucin found on many different types of solid tumors, and a truncated O-glycoform of MUC1, termed Tn-MUC1, has been a target in several immunotherapy strategies [48], including in a vaccine used in human MUC1-expressing transgenic mice [49] as well as in rhesus macaques and humans [50]. In mice, Tn-MUC1 was found to activate glycopeptide-specific CD4+ T cells through antigen presentation on MHC II by dendritic cells or B cells, demonstrating that glycoforms of an MHC-presented peptide can be recognized through TCR interaction. In humans, Tn-MUC1-specific CD4+ and CD8+ T-cells were found to be present in 5 out of 7 patients vaccinated with Tn-MUC1-loaded dendritic cells. Similarly, O-GlcNAc-specific T-cell responses have been observed against shared O-GlcNAc peptides identified through immunoglycoproteomics of leukemias. These peptides were presented by MHC class I molecules and an O-GlcNAc-specific T-cell line could kill autologous cells pulsed with O-GlcNAc peptide, but not cells pulsed with unmodified peptide. Taken together, these studies indicate that post-translational modifications of peptides, especially O-linked modifications, may represent a novel class of neoantigens for TCR-based immunotherapy.

Altered glycosylation on the membrane of malignant cells is a common characteristic of cancer [51]. This change in post-translational modifications increases the number of tumor-specific antigens for CAR T-cell binding. The first CAR to take advantage of these glycosylation differences was directed against tumor-associated glycoprotein (TAG-72), a truncated sialyl-Tn O-glycan located on the cell surface of O-glycoproteins [52] known to be overexpressed by epithelial adenocarcinomas [53]. Designed as a first-generation CAR, CC49 CAR T-cells were able effectively target gastrointestinal tumor lines expressing TAG-72. The first human trial of TAG-72 CAR T-cells led to a significant decrease of both serum TAG-72 levels as well as a decrease in serum CEA levels. Despite this changes, no clinical response was attained [54], likely due to the lack of T cell proliferation as well as rejection due to immunogenicity against the CC49 scFv. More recent studies focused on targeting TAG-72 include the development of a second-generation CAR that shared the same CC49 scFv and added a 4-1BB costimulatory domain for enhanced T-cell survival. The second-generation TAG-72 CAR T-cells showed positive tumor killing in mouse models [55]. Another study evaluated a CAR targeting both TAG-72 as well as the macrophage suppressive tumor marker CD47 [56]. This study showed that CAR T-cells with the ability to bind both markers were able to clear target cells in vitro and may be able to reduce the chance of antigen-loss relapses in human patients.

Another example of a differentially glycosylated tumor antigen is the large mucin protein mucin 1 (MUC1), which is heavily O-glycosylated and often expresses truncated O-glycans, such as Tn antigen, in tumor cells. The monoclonal antibody 5E5 is able to selectively target the Tn-glycoform of MUC1 [48]. Using the variable domains of the 5E5 antibody as an scFv, a second-generation, 4-1BB-costimulated CAR generated robust anti-tumor activity in cell-line derived xenograft models of human T-cell leukemia and metastatic pancreatic cancer. A phase I clinical trial evaluating CAR T-cells targeting Tn-MUC1 in several clinical indications began in 2019 (NCT04025216).

Lewis Y (LeY) is another clinically relevant oligosaccharide that is a promising target for CAR T-cells. While the function of LeY is unknown, it is presented on a number of proteins at a high copy number, including some tumor-associated antigens [57]. A second-generation CAR with a CD28 costimulatory domain demonstrated preclinical efficacy by targeting of the LeY antigen in mice bearing subcutaneous OVCAR3 ovarian cancer tumors [58] and a clinical trial was opened to determine the efficacy in patients with acute myeloid leukemia (AML). One patient achieved a transient CR and two patients achieved a PR. However, disease progressed in all five patients and the best response of 23 months until progression was associated with increased CAR T-cell persistence. Another trial was initiated in 2019 in Australia and is ongoing at the time of this review.

Another CAR design that allows targeting of glycans utilizes natural glycan-binding proteins or lectins as the extracellular antigen-specific domain. A study by Meril et al. developed CARs incorporating the exodomains of human Siglec-7 and Siglec-9 to bind cognate sialoglycans [59]. Siglec-based CAR T-cells were able to mediate antitumor activity against cell lines derived from cancer histotypes as varied as leukemia and ovarian cancer in vitro as well as a patient-derived melanoma xenograft model in NSG mice. This use of human receptors or ligands as the binding domain of CAR T-cells may reduce the immunogenicity of chimeric protein-expressing cell therapies, such as the human anti-mouse reactivity observed in some clinical CAR T-cell studies due to the recognition of murine-based scFvs.

## 5. Phospho-Antigens

Surface phospho-antigens can also be recognized by circulating T-cells. This recognition is limited to a very specific subset of T-cells defined by the expression of γδ TCRs, specifically expressing Vδ2 as well as Vγ9 (Vγ9Vδ2 T-cells). These T-cells recognize phosphoantigens presented by butyrophilin 3A (BTN3A) molecules [60] or butyrophilin 2A (BTN2A) [61].

Butryophilin molecules are genes that are required for stimulation of Vγ9Vδ2 T-cells by phosphoantigens and are related to the B7 family of proteins, which comprise co-stimulatory and co-inhibitory molecules [62]. There are three subfamilies of butyrophilin molecules (BTN1, BTN2, and BTN3), with the most homology existing between BTN2A and BTN3A. BTN3A molecules have been controversial in the way that they present antigen and stimulate Vγ9Vδ2 T-cells. BTN3A is a subfamily made up of three genes: BTN3A1, BTN3A2, and BTN3A3. A recent study defined the importance of an intracellular B30.2 domain, which is part of BTN3A1. This B30.2 domain was found to be the stimulatory domain for Vγ9Vδ2 T-cells after it was chimerically added to BTN3A3, a protein typically characterized as non-stimulatory [63]. After engraftment of the B30.2 domain from BTN3A1 onto BTN3A3, the domain was able to stimulate Vγ9Vδ2 T-cells.

γδ T-cells are a smaller population of T-cells defined by their differences in TCR that separates them from αβ T-cells. These cells, fittingly, are made of TCRs that contain a γ chain and a δ chain as opposed to the traditional α and β chains. Vγ9Vδ2 T-cells, when activated, are able to exert a range of different effector functions, including the killing of infected cells [64]. This subset of cells constitutes 1–5% of total circulating T-cells. However, during infections, the subset is increased in frequency to over 50% [65]. This subset of cells often express CD45RO at a high frequency, leading to a more memory-like phenotype. This leads to a more innate-like T-cell response, as opposed to an acquired effector-like response. While only 1–5% of total T-cells are Vγ9Vδ2 T-cells, over 1 in 40 total memory T-cells are Vγ9Vδ2 T-cells [66]. This phenotype allows Vγ9Vδ2 T-cells to target a large number of phosphoantigens instead of specifically binding to only one. Upon activation with phosphoantigen, Vγ9Vδ2 T-cells preferentially differentiate into a Th1-like phenotype, characterized by high IFN-γ and TGF-β production [67]. However, they can also be induced into Th2, Th17, and Treg populations according to the cytokine profile presented to them. For example, Th2 differentiation happens with IL-4 stimulation and Th17 differentiation happens with stimulation with IL-1α, IL-23, and TGFβ [68].

Vγ9Vδ2 T-cells are known to have both a pro and anti-immunogenic effect on tumors. In vitro and in mouse models, they have been shown to be cytotoxic against many different types of tumor lines [69]. Cytotoxic activity against tumors is characterized by IFN-γ and TNF-α release as well as an increase in granzyme and perforin production [68]. However, protumor activity is also found with Vγ9Vδ2 T-cells. Vγ9Vδ2 T-cells are known to suppress CD4+ T-cell proliferation as well as produce anti-inflammatory cytokines such as IL-10, suggesting that a population of these cells have a regulatory or suppressive phenotype [70].

While some αβ T-cells are also able to target phosphoantigens through the TCR, the majority of cellular phosphorylation occurs intracellularly, which has likely limited pursuit of this class of targets for CAR development. The topic of phosphorylation on the cell membrane is debated. However, studies have shown that extracellular phosphorylation by secreted kinases does exist [71]. This phosphorylation can lead to biological effects, such as the phosphorylation of the receptor tyrosine kinase EphB2, leading to interactions between EphB2 and N-methyl-D-asparate receptors (NMDARs), in turn leading to pain [72]. Certain types of cancer have also shown an increase in extracellular phosphorylation. A major increase in ecto-protein kinase (ecto-PKA) has been shown in the serum of breast cancer patients as well as increases of PKA, PKC, and CK2 in prostasomes in prostate cancer, which may lead to an increase in extracellular phosphorylation [73]. If these modifications are common, then extracellular phosphates should be studied as a possible new class of targets for CAR T-cells. No studies currently exist evaluating phosphoantigen-specific CARs.

## 6. Potential Targets for CAR T-Cells: GlycoRNA

A recent paper by Flynn et al. has described glycosylated RNA present on the membrane of different types of cells [74]. GlycoRNAs are trafficked to the cell membrane and can contain sialoglycans that are recognizable by sialic acid binding immunoglobulin lectin-type (Siglec) receptors Siglec-11 and Siglec-14. Siglecs are sialoglycan-binding immune receptors with roles in inhibition of immune activation, akin to the role of PD1 in T-cells. While the identification of glycoRNA is recent, this discovery should encourage the investigation into whether glycoRNA is selectively or more abundantly expressed by tumor cells. In addition, CAR molecules can be developed to target cell surface RNA and glycoRNA, which is yet another example of the expansion of targetable antigens CAR molecules have added to the cellular immunity toolbox.

Other potential targets for CAR T-cell therapies have been reviewed in the past [75,76].

## 7. Conclusions

T-cell recognition of different types of biomolecules continues to be an important aspect of immunology research. While naturally occurring T-cells are able to recognize many types of antigen, their targeting is restricted to antigen presentation on domains such as MHC, CD1, and BTN. The addition of a chimeric receptor to T-cells allows the targeting of membrane-bound antigens that were previously un-targetable by T-cells. CAR T-cells have proven to be an effective tool in the fight against cancer by allowing the MHC-independent targeting of tumor-specific surface molecules. Further research continues to improve upon the types of targets for CAR T-cells as well as improving on CAR designs.

## Data Availability

Not applicable.
